# Association of Image-Guided Navigation With Complete Resection Rate in Patients With Locally Advanced Primary and Recurrent Rectal Cancer: A Nonrandomized Controlled Trial

**DOI:** 10.1001/jamanetworkopen.2020.8522

**Published:** 2020-07-08

**Authors:** Esther N. D. Kok, Ruben van Veen, Harald C. Groen, Wouter J. Heerink, Nikie J. Hoetjes, Erik van Werkhoven, Geerard L. Beets, Arend G. J. Aalbers, Koert F. D. Kuhlmann, Jasper Nijkamp, Theo J. M. Ruers

**Affiliations:** 1 Department of Surgical Oncology, the Netherlands Cancer Institute—Antoni van Leeuwenhoek, Amsterdam, the Netherlands; 2 Department of Biometrics, the Netherlands Cancer Institute—Antoni van Leeuwenhoek, Amsterdam, the Netherlands; 3 Faculty Applied Sciences, Group Nanobiophysics, Twente University, Enschede, the Netherlands

## Abstract

**Importance:**

The percentage of tumor-positive surgical resection margin rates in patients treated for locally advanced primary or recurrent rectal cancer is high. Image-guided navigation may improve complete resection rates.

**Objective:**

To ascertain whether image-guided navigation during rectal cancer resection improves complete resection rates compared with surgical procedures without navigation.

**Design, Setting, and Participants:**

This prospective single-center nonrandomized controlled trial was conducted at the Netherlands Cancer Institute—Antoni van Leeuwenhoek in Amsterdam, the Netherlands. The prospective or navigation cohort included adult patients with locally advanced primary or recurrent rectal cancer who underwent resection with image-guided navigation between February 1, 2016, and September 30, 2019, at the tertiary referral hospital. Clinical results of this cohort were compared with results of the historical cohort, which was composed of adult patients who received rectal cancer resection without image-guided navigation between January 1, 2009, and December 31, 2015.

**Intervention:**

Rectal cancer resection with image-guided navigation.

**Main Outcomes and Measures:**

The primary end point was the complete resection rate, measured by the amount of tumor-negative resection margin rates. Secondary outcomes were safety and usability of the system. Safety was evaluated by the number of navigation system–associated surgical adverse events. Usability was assessed from responses to a questionnaire completed by the participating surgeons after each procedure.

**Results:**

In total, 33 patients with locally advanced or recurrent rectal cancer were included (23 men [69.7%]; median [interquartile range] age at start of treatment, 61 [55.0-69.0] years). With image-guided navigation, a radical resection (R0) was achieved in 13 of 14 patients (92.9%; 95% CI, 66.1%-99.8%) after primary resection of locally advanced tumors and in 15 of 19 patients (78.9%; 95% CI, 54.4%-94.0%) after resection of recurrent rectal cancer. No navigation system–associated complications occurred before or during surgical procedures. In the historical cohort, 142 patients who underwent resection without image-guided navigation were included (95 men [66.9%]; median [interquartile range] age at start of treatment, 64 [55.0-70.0] years). In these patients, an R0 resection was accomplished in 85 of 101 patients (84.2%) with locally advanced rectal cancer and in 20 of 41 patients (48.8%) with recurrent rectal cancer. A significant difference was found between the navigation and historical cohorts after recurrent rectal cancer resection (21.1% vs 51.2%; *P* = .047). For locally advanced primary tumor resection, the difference was not significant (7.1% vs 15.8%; *P* = .69). Surgeons stated in completed questionnaires that the navigation system improved decisiveness and helped with tumor localization.

**Conclusions and Relevance:**

Findings of this study suggest that image-guided navigation used during rectal cancer resection is safe and intuitive and may improve tumor-free resection margin rates in recurrent rectal cancer.

**Trial Registration:**

Netherlands Trial Register Identifier: NTR7184

## Key Points

**Question:**

Is there an association between image-guided navigation and complete surgical resection rates in locally advanced rectal cancer and recurrent rectal cancer?

**Findings:**

In this nonrandomized controlled trial of 33 patients with locally advanced or recurrent rectal cancer, image-guided navigation was found to be a feasible and safe technique in advanced rectal cancer resection. Patients with recurrent cancer who underwent image-guided resection had higher rates of successful resection than a historical cohort who received resection without navigation; however, there was no difference between groups for patients with primary locally advanced cancer.

**Meaning:**

Image-guided navigation appeared to be associated with an increase in radical resection margin rates in recurrent rectal cancer resection and thereby may improve patient outcomes.

## Teaser

This nonrandomized controlled trial evaluates the resection margin rates as well as the safety and usability of a newly developed image-guided navigation technique among patients who underwent a rectal cancer resection.

## Introduction

Surgical resection is the main treatment for patients with rectal cancer. This procedure can be challenging, especially in patients with primary advanced disease in which the mesorectal fascia is threatened and tissue planes are disrupted by tumor ingrowth, fibrosis, or radiation. Despite improved neoadjuvant treatment and refinement of surgical techniques, the proportion of tumor-positive resection margin rates remains 10% to 15%.^1,2^ Previous studies have shown that a positive resection margin rate is associated with high local recurrence and low survival rates.^3,4^ Surgical resection in patients with local recurrence is even more challenging because of fibrotic scar tissue of the previous operation. Rectal cancer recurrence is often accompanied by tumor ingrowth in surrounding tissue and organs, requiring extensive surgical procedures such as pelvic exenteration. These procedures are associated with high tumor-positive resection margin rates of 38% to 62%, major complication rates of 32% to 60%, and 30-day mortality rates of 0% to 16%.^5,6,7,8,9,10,11^

A possible means to improve the safety and effectiveness of surgical procedures for locally advanced and recurrent rectal cancer could be image-guided navigation. This technique provides surgeons with real-time intraoperative feedback on the position of surgical instruments in reference to the intraoperative anatomy, including tumor borders. As such, the technique could improve the complete removal of tumors without damaging the surrounding healthy structures.^12^

In an investigation of whether image-guided navigation was possible in more advanced rectal resection, a novel electromagnetic surgical navigation system was developed for pelvic malignant neoplasms. This system was shown to be safe, feasible, and accurate.^13^ In the current study, we evaluated the clinical performance of this image-guided navigation technique. We hypothesized that surgical navigation during advanced and recurrent rectal cancer resection would allow for the full use of preoperative imaging during the surgical procedure and would improve surgical outcome by a higher complete resection rate compared with results in a historical cohort. Moreover, we examined the safety and usability of this intraoperative technique.

## Methods

### Study Design and Patient Selection

We conducted a prospective, single-center nonrandomized controlled trial at the Netherlands Cancer Institute—Antoni van Leeuwenhoek, a tertiary referral hospital in Amsterdam, the Netherlands, to evaluate the use of an electromagnetic navigation system during abdominal surgical procedures. This trial consisted of individuals with locally advanced primary or recurrent rectal cancer who underwent a resection with image-guided navigation between February 1, 2016, and September 30, 2019. This study was approved by the institutional review board of the Netherlands Cancer Institute—Antoni van Leeuwenhoek. All patients provided written informed consent. We followed the Transparent Reporting of Evaluations with Nonrandomized Designs (TREND) reporting guideline.

**Figure 1.**
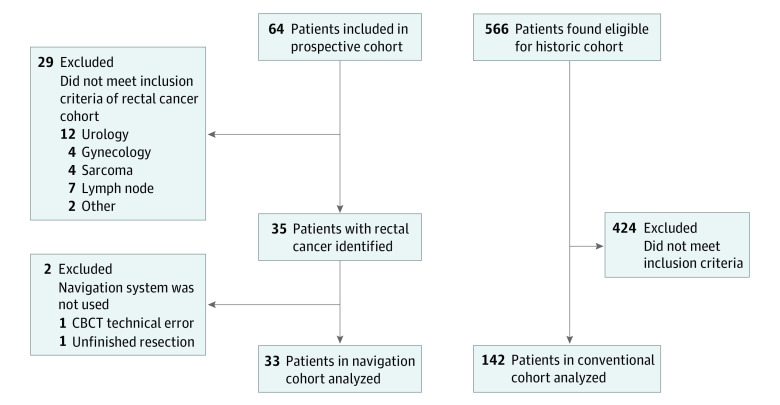
Study Flow Diagram of the Prospective and Historical Cohorts CBCT indicates cone-beam computed tomography.

The inclusion criteria for the prospective cohort were as follows: diagnosis of either locally advanced primary or recurrent rectal cancer for which a radical resection was judged by surgeons as challenging, patient age of 18 years or older, suitability for contrast-enhanced computed tomography scanning, and scheduled abdominal surgical procedure at the Netherlands Cancer Institute—Antoni van Leeuwenhoek. Patients with metal pelvic implants or with a pacemaker were excluded because they could alter the preoperative imaging. Locally advanced primary rectal cancer was defined as T3 or T4 tumors extending close to (<2 mm) or invading the mesorectal fascia, as shown on rectal magnetic resonance imaging. Recurrent rectal cancer was defined as cancer that recurred in the pelvic area after earlier treatment. The inclusion and exclusion criteria are reported in the trial protocol (Supplement 1), and the patient selection diagram is shown in Figure 1.

The prospective or navigation cohort who underwent a resection with image-guided navigation was compared with a historical or conventional cohort. Patients with locally advanced primary or recurrent rectal cancer; who were aged 18 years or older; and who received rectal resection without the navigation system between January 1, 2009, and December 31, 2015, were eligible for inclusion in the historical cohort. Clinical data were collected retrospectively from medical records and then anonymized. The historical group was well balanced with the navigation group, and no significant differences in baseline characteristics were found between the 2 cohorts. Permission to collect the retrospective data was granted by the institutional review board of the Netherlands Cancer Institute—Antoni van Leeuwenhoek.

### Image-Guided Navigation 

**Figure 2.**
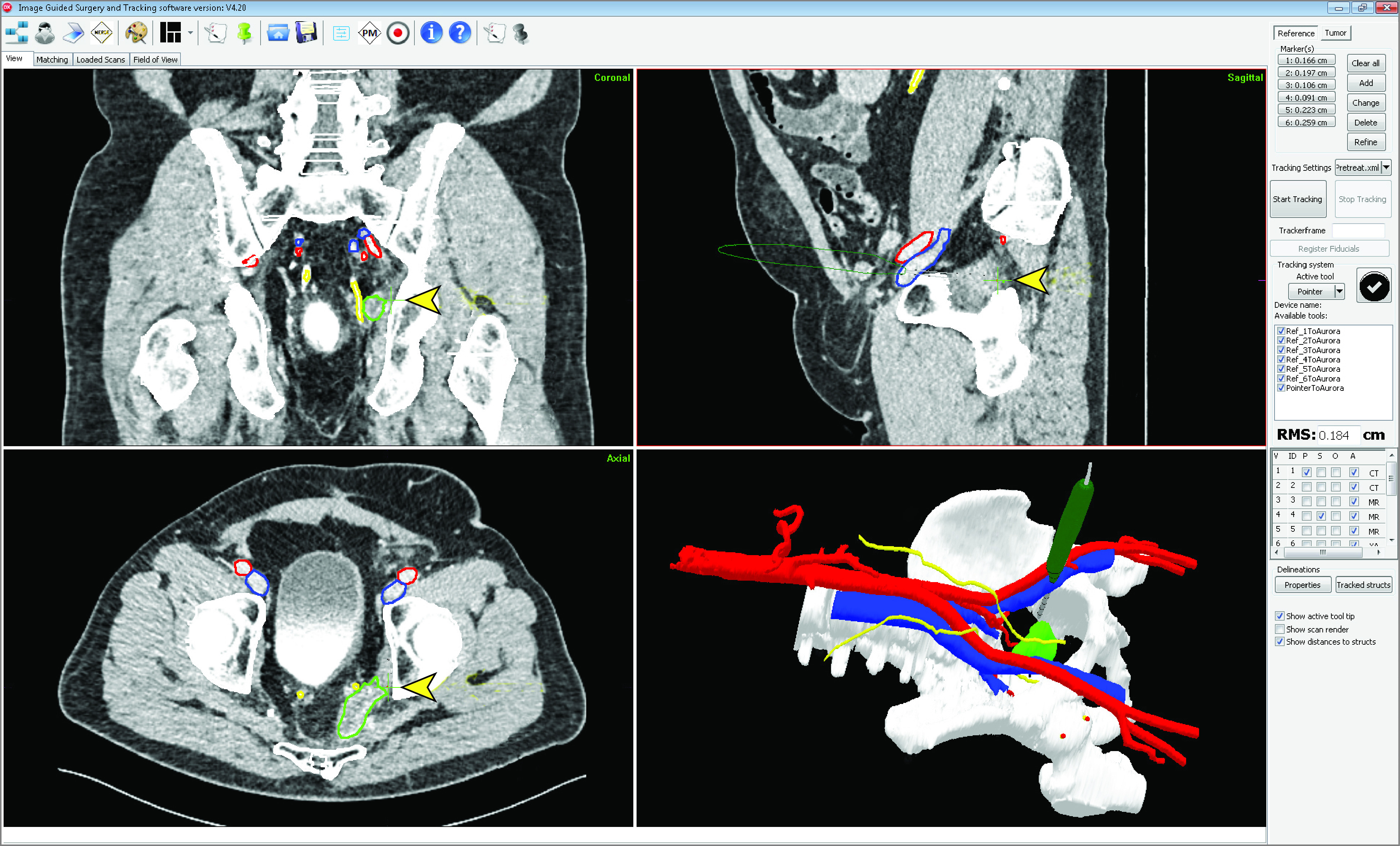
Example of the Image-Guided Navigation System Interface During Recurrent Rectal Cancer Resection The planning computed tomography scan, with segmentations in the coronal, sagittal, and axial planes, and the 3-dimensional model are shown. The surgical pointer is shown in the 3-dimensional model and orthogonal views (highlighted with arrowheads). The tumor is segmented in green, the ureters in yellow, and the vessels in blue/red.

Full details of the navigation setup have been published elsewhere.^13^ Briefly, a patient-specific 3-dimensional model based on preoperative imaging (computed tomography and magnetic resonance imaging) was created. In the operating room, a cone-beam computed tomography scan of the patient in surgical position was acquired. The intraoperative images were registered to the preoperative images based on the bony structures. An electromagnetic tracking system constantly linked the preoperative images and 3-D model to the intraoperative position of the patient by electromagnetic sensors affixed on the skin. During the surgical procedure, an electromagnetic tracked pointer was available to validate the registration accuracy and to assess the anatomy and tumor location (Figure 2). No additional monitoring or auditing visits were required after the operation.

### Study End Points 

The primary end point of this study was the complete resection rate, measured by the number of tumor-negative resection margin rates. After primary rectal cancer resection, a tumor larger than 1 mm of the circumferential resection margin rate was defined as radical (R0). After recurrent rectal cancer resection, the resection margin rate was defined as radical (R0) when no tumor cells reached the border of the inked resection specimen.

The secondary end points were safety and usability of the system. Safety was evaluated by the number of navigation system–associated surgical adverse events. Intraoperative surgical complications not associated with the navigation system were also reported. Usability was assessed from responses to a questionnaire completed by the participating surgeons after each procedure.

The questionnaire consisted of 2 parts. The first part tested surgical usability with the 10-item System Usability Scale (SUS), with 5 response options ranging from strongly disagree to strongly agree. The 10 questions on the SUS can result in a total score of 0 to 100 points, representing the surgeon’s level of satisfaction with the novel technique (ie, from highly unusable to highly usable^14^). An SUS score of 70 points or higher is considered good, with a high chance of acceptance of the technology by the surgeons.^15^ The second part of the questionnaire compared the effectiveness (survival, complications, and resection margin rates), efficiency (extra time and tumor localization), and decisiveness during the resection between the conventional setting and the image-guided navigation setting. A 5-point Likert scale was used for each question, and a score higher than 3 points was in favor of the navigation setting. In addition, the required time to set up the navigation system, including positioning of the patient trackers and acquiring the intraoperative cone-beam computed tomography imaging, was reported.

### Statistical Analysis

No sample size calculations were performed. All analyses were performed in SPSS, version 25.0 (IBM Corp). Patient characteristics were presented as number (%) or median (interquartile range [IQR]). Categorical variables were compared using a Fisher exact test, and continuous variables were compared with the Mann-Whitney test. The 95% CIs were calculated with the Clopper Pearson method. A logistic regression model was used to ascertain whether the association of the navigation system with surgical resection margin rates remained statistically significant after adjusting for neoadjuvant therapy. A 2-sided *P* ≤ .05 was considered statistically significant. Data analysis was conducted between November 11, 2019, and January 21, 2020.

## Results

In total, 64 patients were included in the prospective cohort (Figure 1); of these patients, 33 (23 men [69.7%]; median [IQR] age at start of treatment, 61 [55.0-69.0] years) met the inclusion criteria and were analyzed. Fourteen of 33 patients (42.4%) had locally advanced primary rectal cancer, and 19 (57.6%) had recurrent rectal cancer (Table). In all 33 patients, image-guided navigation was used without technical failure. The mean (SD) required time to set up the navigation system in the operating room was 16 (6.0) minutes.

**Table.** Patient Characteristics

Characteristic	No. (%)		*P* value
	Navigation cohort (n = 33)	Historical control cohort (n = 142)	
**Locally advanced primary rectal cancer**			
Total	14 (42.4)	101 (71.1)	
Sex			
Men	12 (85.7)	73 (72.3)	.35
Women	2 (14.3)	28 (27.7)	
Age at start of treatment, median (IQR), y	58.0 (35-71)	61.0 (25-82)	.49
Clinical tumor and nodal stage			
T3N0-2 MRF+	7 (50)	62 (61.4)	.56
T4N0-2 MRF+	7 (50)	39 (38.6)	
Distant metastases			
Present	3 (21.4)	24 (23.8)	>.99
Absent	11 (78.6)	77 (78.6)	
Primary tumor’s location from anorectal verge, cm			
Low (0-5)	10 (71.4)	52 (51.5)	.46
Middle (5-10)	3 (21.4)	31 (30.7)	
High (10-15)	1 (7.1)	18 (17.8)	
Neoadjuvant treatment			
None	0	1 (1.0)	.13
Short-course radiotherapy (5 × 5 Gy)	0	3 (3.0)	
Chemoradiation	8 (57.2)	80 (79.2)	
5 × 5 Gy + chemotherapy	5 (35.7)	16 (15.8)	
Chemoradiation + chemotherapy	1 (7.1)	1 (1.0)	
Type of surgical resection			
APR			.10
Open	2 (14.3)	38 (37.6)	
Laparoscopic	0	3 (3.0)	
LAR			
Open	12 (78.6)	35 (34.7)	
Laparoscopic	1 (7.1)	10 (9.9)	
Exenteration	0	15 (14.9)	
Pathological outcome			
ypT0N0	2 (14.3)	5 (5.0)	.39
ypT2N0-2	2 (14.3)	8 (7.9)	
ypT3N0	2 (14.3)	35 (34.7)	
ypT3N1	3 (21.4)	14 (13.9)	
ypT3N2	4 (28.6)	28 (27.7)	
ypT4N0	0	7 (6.9)	
ypT4N1	1 (7.1)	1 (3.0)	
ypT4N2	0	1 (1.0)	
**Recurrent rectal cancer**			
Total	19 (57.6)	41 (28.9)	
Sex			
Men	11 (57.9)	22 (53.7)	.79
Women	8 (42.1)	19 (46.3)	
Age at start of treatment, median (IQR), y	61.5 (52-78)	67.0 (41-82)	.08
Recurrent tumor location			
Pelvic wall/presacral	14 (73.7)	26 (63.9)	.56
Staple line recurrence	5 (26.3)	15 (36.6)	
Neoadjuvant treatment			
None	0	5 (12.2)	.06
Short-course radiotherapy	0	2 (4.9)	
Chemotherapy	1 (5.3)	2 (4.9)	
Chemoradiation	11 (57.9)	29 (70.7)	
Chemoradiation + chemotherapy	7 (36.8)	3 (7.3)	
Type of surgical resection			
Open APR	3 (15.8)	19 (46.3)	.10
Open LAR	4 (21.1)	6 (14.6)	
Exenteration	8 (42.1)	14 (34.2)	
Local	4 (20.0)	2 (4.9)	

Abbreviations: APR, abdominoperineal resection; IQR, interquartile range; LAR, low anterior resection; MRF, mesorectal fascia.

A total of 142 patients were included in the historical cohort and analyzed. Of these patients, 95 were men (66.9%) and the median (IQR) age at start of treatment was 64 [55.0-70.0] years. Most patients (101 [71.1%]) had locally advanced primary rectal cancer, and 41 (28.9%) had recurrent rectal cancer (Table).

At the Netherlands Cancer Institute—Antoni van Leeuwenhoek, 85 patients with locally advanced primary or recurrent rectal cancer underwent surgical resection. The navigation system was not used in 52 patients mainly because surgeons considered radical resection less challenging or because of logistical reasons (eg, no available navigation system or hybrid operating room).

With image-guided navigation, a radical resection (R0) was accomplished in 13 of 14 prospective patients (92.9%; 95% CI, 66.1%-99.8%) with locally advanced rectal cancer. A radical resection after recurrent rectal cancer resection was accomplished in 15 of 19 prospective patients (78.9%; 95% CI, 54.4%-94.0%). In the historical cohort, an R0 resection was accomplished in 85 of 101 patients (84.2%; 95% CI, 75.6%-90.7%) with locally advanced rectal cancer and in 20 of 41 patients (48.8%; 95% CI, 32.9%-64.9%) with recurrent rectal cancer.

### Safety and Usability of the System

No navigation system–associated complications occurred before or during rectal resection in patients with locally advanced primary or recurrent rectal cancer. In patients with locally advanced primary rectal cancer, no intraoperative complications were observed. In patients with recurrent rectal cancer, intraoperative complications were reported in 4 patients: 1 had a ureter injury and 3 had intra-abdominal bleeding during the resection. None of these complications could be attributed to the use of image-guided navigation.

**Figure 3.**
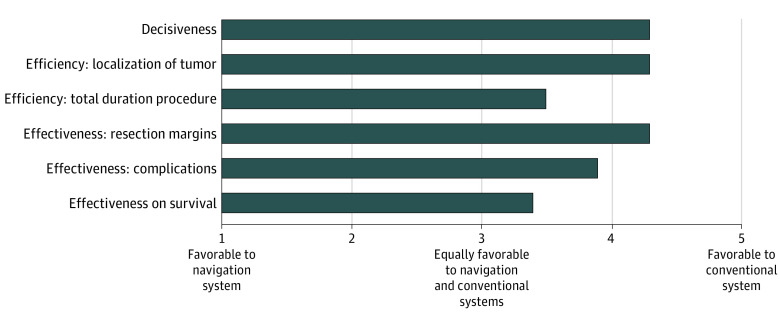
Comparison Between Navigation and Conventional Systems

Nine surgeons performed a resection with the navigation system and completed 21 questionnaires in total. Eighteen of all 21 of these procedures (85.7%) were classified by the surgeons as complex. Surgeons stated that the navigation system simplified the procedure for all complex cases. The navigation technique was scored with a median (IQR) SUS score of 75.0 (42-87) points, which indicated a high level of usability with a great chance of acceptance of the technique (eFigure in Supplement 2). In 18 of 21 completed questionnaires (85.7%), the surgeons reported wanting to use the technology during future procedures. The questions comparing the conventional setting with the navigation setting were given a mean (SD) score of 3.9 (0.4) points, indicating that surgeons preferred the navigation over the conventional setting. Questions about tumor localization (mean [SD] score, 4.3 [3.44-5.11]), securing resection margin rates, and decisiveness scored high in favor of the navigation setting (mean [SD] score for both, 4.3 [3.59-4.94]) (Figure 3).

### Comparison of Navigation and Historical Cohorts

**Figure 4.**
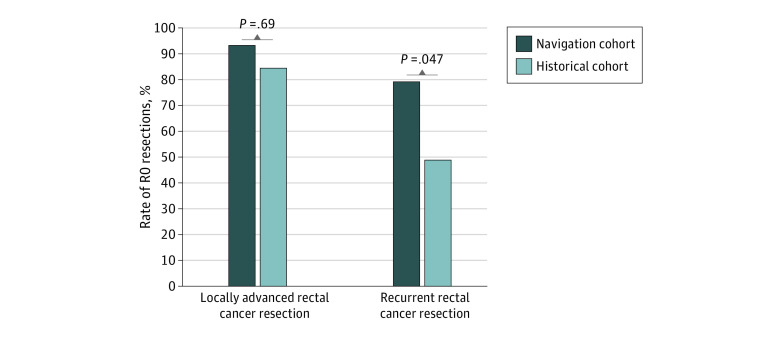
Comparison of Complete Resection Rates Between the Navigation and Historical Cohorts

After primary tumor resection for locally advanced rectal cancer, a complete resection (R0) was accomplished in 85 of 101 patients in the historical cohort (84.2%; 95% CI, 75.6%-90.7%) and in 13 of 14 patients in the navigation cohort (92.9%; 95% CI, 66.1%-99.8%). After resection of recurrent rectal cancer, an R0 resection was achieved in 20 of 41 patients in the historical cohort (48.8%; 95% CI, 32.9%-64.9%) and in 15 of 19 patients in the navigation cohort (78.9%; 95% CI, 54.4%-94.0%). Patients in the navigation cohort showed significantly lower tumor-positive margin rates after recurrent rectal cancer resection compared with patients in the historical cohort (21.1% vs 51.2%; *P* = .047). For the locally advanced rectal cancer resection, the difference was not significant (7.1% vs 15.8%; *P* = .69) (Figure 4).

In the historical cohort, intraoperative complications were observed in 5 patients after primary tumor resection: ureter injury (n = 2), intra-abdominal bleeding (n = 2), and spleen injury (n = 1). After recurrent rectal cancer resection, intraoperative complications occurred in 6 patients: abdominal bleeding (n = 2) and ureter injury (n = 4). No significant differences in intraoperative complications were found between the navigation and historical cohorts.

When comparing the prospective cohort to the historical cohort, no significant difference was observed between the 2 groups (Table). Despite this finding, we analyzed the potential implication of the less-balanced characteristic, neoadjuvant chemotherapy. After controlling for neoadjuvant therapy in a logistic regression model for patients with recurrent rectal cancer, the use of image-guided navigation was associated with complete resection margin rates (odds ratio [OR], 4.10; 95% CI, 1.07-15.78; *P* = .04). After locally advanced primary tumor resection, the use of image-guided navigation was not associated with complete resection margin rates after controlling for neoadjuvant therapy (OR, 2.46; 95% CI, 0.29-20.72; *P* = .41). Because of a possible selection bias owing to the restrictive inclusion by the surgeons, we did not analyze the patient cohort for whom image-guided navigation was not used between February 1, 2016, and September 30, 2019. Baseline characteristics of patients who underwent resection with navigation vs those without navigation differed considerably in cTNM and ypTNM staging (all in favor of the patients who received rectal resection without navigation), which would make any comparison invalid.

## Discussion

Patients with locally advanced primary or recurrent rectal cancer who underwent a surgical procedure with image-guided navigation showed high complete resection rates. The system was demonstrated to be safe, and no navigation system–associated complications were observed. In patients with recurrent rectal cancer, image-guided resection was associated with increased resection margin rates, even after adjustment for neoadjuvant therapy. Surgeons rated the navigation setting more favorably vs the conventional setting and stated that the technique improved decisiveness and simplified complex procedures.

Image-guided navigation in pelvic surgical procedures has been described previously.^13,16,17,18,19^ Two separate groups investigated the feasibility of navigation in pilot studies for Transanal Minimal Invasive Surgery–Total Mesorectal Excision (TAMIS-TME).^16,17,18,19^ Both groups concluded that navigation was feasible and safe, with satisfactory accuracy for clinical use. Although promising results were shown, only small patient cohorts of 1 to 3 patients were evaluated. One study tested image-guided navigation in a large group.^13^ Outcomes of this pilot study were encouraging, showing the navigation system was safe, feasible, and accurate (mean target registration error: 4.0 mm). However, the study included a wide variety of pelvic tumors and mainly focused on the feasibility and accuracy of the navigation system.^13^ The current study aimed to evaluate the clinical outcomes of surgical navigation specifically in patients with rectal cancer. To our knowledge, this is the first study to compare the resection margin rates of patients receiving rectal resection with or without image-guided navigation.

In this study, a significant difference was found in surgical resection margin rates between the navigation and historical cohorts in patients with recurrent rectal cancer. The positive resection margin rate in the historical control cohort was in line with the margin rates in other studies, varying between 38% and 62%.^9,10,20^ With image-guided navigation, the percentage of positive resection margin rate was 21.1%. In patients with locally advanced primary rectal cancer, no significant difference was found between the navigation and the historical cohorts. This similarity could be explained by the generally much lower positive resection margin rates in primary rectal cancer resection (7.1%) and by the small sample size of the navigation cohort (n = 33). Literature on positive resection margin rates in rectal cancer often considers all different stages of rectal cancer. Data on the positive resection margin rate for locally advanced primary rectal cancer are scarce, and the rates range from 9% to 34%.^1,9,10,21^ The study by Rickles et al^1^ included 8044 patients with cT3 rectal tumors and reported tumor-positive resection margin rates in 17.0% of the patients; it also involved 773 patients with cT4 rectal tumors and showed tumor-positive resection margin rates in 35.2% of the patients. The results of the historical cohort in the current study, with 15.8% positive resection margin rate, are in line with results reported by Rickles et al.^1^ The 7.1% positive resection margin rate in the navigation group, although not statistically different from the margin rate in the historical cohort, compares favorably with findings in Rickles et al.^1^

Integration of new surgical techniques requires the evaluation of safety and usability. In this study, no navigation system–associated complications were observed, a finding that is consistent with previous results.^13^ The usability of the system was given an SUS score of 75 points, and surgeons preferred the navigation setting over the conventional setting in all questions. Moreover, the time required for setting up the navigation system in this study was limited compared with the time reported in other studies, in which setup required 25 to 47 minutes.^17,19^ All of these results suggest that the navigation system that we analyzed is safe and intuitive with a high chance of clinical acceptance outside of the research setting.

### Limitations

This study has some limitations. First, the nonrandomized trial design used to compare a prospective cohort with a historical cohort has inherent methodological shortcomings. However, the historical control group that we selected was well balanced with the navigation cohort, with no differences in baseline characteristics. Nonetheless, relevant differences in the characteristics existed between the 2 groups, although none of them were statistically significant. Therefore, even though the analysis plan did not specify any adjusted analysis, we decided to adjust for neoadjuvant therapy. Second, this was a single-center study with small sample sizes in the navigation cohort. Third, a dedicated clinical implementation team that gave technical support was highly involved, and it remains unclear whether the navigation system would generate the same results if transferred to other hospitals. Fourth, the resection margin rates could have been altered by the performance of the individual surgeons and the resulting quality of the total mesorectal excision specimen. Because of the retrospective setup of the historical cohort and the small sample size, these variables could not be taken into account. Nevertheless, the implications may be limited given that, in our institution, resections are performed by a team of 2 experienced surgeons in rectal cancer, and the results of the mesorectal resection specimen are discussed on a weekly basis in a multidisciplinary meeting.

## Conclusions

Image-guided navigation appeared to be safe and was associated with an increase in radical resection margin rates in recurrent rectal cancer resection. To our knowledge, this study was the first to compare resection margin rates in patients with rectal cancer who underwent resection with image-guided navigation and those who received resection without navigation. Participating surgeons gave high ratings to the improved decisiveness regarding resection margin rates and tumor localization associated with the navigation technique.

## Notes

Supplement 1. **Trial Protocol**

Supplement 2. **eFigure.** Mean Scores of Items in the System Usability Scale
