# Errors in Figure 3 and Supplement 2

**DOI:** 10.1001/jamanetworkopen.2020.18477

**Published:** 2020-08-14

In the Original Investigation titled “Association of Image-Guided Navigation With Complete Resection Rate in Patients With Locally Advanced Primary and Recurrent Rectal Cancer: A Nonrandomized Controlled Trial,”^1^ published July 8, 2020, there were errors in Figure 3, and the eFigure was omitted from Supplement 2. In Figure 3, the label under 1 and 5 should have appeared as “Favorable to conventional system” and “Favorable to navigation system,” respectively. This article has been corrected.
