# Supplementary material for: Association of Image-Guided Navigation With Complete Resection Rate in Patients With Locally Advanced Primary and Recurrent Rectal Cancer: A Nonrandomized Controlled Trial
Source: JAMA Netw Open. 2020 Jul 8;3(7):e208522. doi: 10.1001/jamanetworkopen.2020.8522 (PMC7344384; doi:10.1001/jamanetworkopen.2020.8522)
Supplement: Supplement 1. — Trial Protocol [file jamanetwopen-3-e208522-s001.pdf]

Image-guided navigation during abdominal surgery

(Navigation 1 study)

Version 8.0

October 3, 2018

PROTOCOL TITLE: ‘*Image-guided navigation during abdominal surgery*’

**Protocol ID**	
**Short title**	**Image-guided navigation during abdominal surgery**
**Version**	**8.0**
**Date**	**October 3, 2018**
**Principal investigator/project leader**	**Prof. Th. J. M. Ruers, Oncological Surgeon**<br>Head of the Division Surgical Oncology<br>NKI-AVL<br>Plesmanlaan 121, 1066 CX Amsterdam<br>T:+31(0)20 5122538<br>t.ruers@nki.nl
**Co-investigator(s)**	**Dr. K.F.D. Kuhlmann**<br>**Surgeon, Fellow gastrointestinal surgical oncology**<br>NKI-AVL<br>Plesmanlaan 121, 1066 CX Amsterdam<br>T:+31(0)20 5126990<br>k.kuhlmann@nki.nl<br><br>**Dr. J. Nijkamp**<br>Implementation specialist image-guided oncology<br>NKI-AVL<br>Plesmanlaan 121, 1066 CX Amsterdam<br>T:+31(0)20 5121774<br>j.nijkamp@nki.nl<br><br>Dr. H.C. Groen<br>Biomedical engineer implementation team<br>NKI-AVL<br>Plesmanlaan 121, 1066 CX Amsterdam<br>T:+31(0)20 512 1004<br>h.groen@nki.nl
**Independent physician(s)**	**W. Prevoo**<br>Radiologist<br>NKI-AVL<br>Plesmanlaan 121, 1066 CX, Amsterdam<br>T:+31(0)20 5121090<br>w.prevoo@nki.nl
**Independent clinical physicist**	**M. Sinaasappel**<br>Clinical physicist<br>NKI-AVL<br>Plesmanlaan 121, 1066 CX, Amsterdam<br>T:+31(0)20 5121519<br>m.sinaasappel@nki.nl

**Laboratory sites**	**The Netherlands Cancer Institute**<br>NKI-AVL<br>Plesmanlaan 121, 1066 CX Amsterdam<br>The Netherlands

PROTOCOL SIGNATURE SHEET

**Name**	**Signature**	**Date**
**Coordinating Investigator/Project leader:**<br>Prof. dr. T. Ruers, surgeon. Head of the Division Surgical Oncology		

The present study is a close collaboration between the following group and persons at the study site:

- Gastrointestinal Surgeons
- Urologists

TABLE OF CONTENTS

1. INTRODUCTION AND RATIONALE 10
2. OBJECTIVES 13
3. STUDY DESIGN 14
4. STUDY POPULATION 15
4.1 Population (base) 15
4.2 Inclusion criteria 15
4.3 Exclusion criteria 15
4.4 Sample size calculation 15
5. TREATMENT OF SUBJECTS 17
5.1 Study procedure 17
5.2 Investigational products 18
5.3 Use of co-intervention 22
6. METHODS 22
6.1 Main study parameters/endpoints 22
6.2 Randomisation, blinding and treatment allocation 23
6.3 Withdrawal of individual subjects 23
6.4 Premature termination of the study 23
6.5 Monitoring and auditing 23
7. SAFETY REPORTING 24
7.1 Section 10 WMO event 24
7.2 Adverse and serious adverse events 24
7.3 Follow up of adverse and serious adverse events 25
7.4 Data Safety monitoring board (DSMB) 25
8. STATISTICAL ANALYSIS 26
8.1 Descriptive statistics 26
9. ETHICAL CONSIDERATIONS 26
9.1 Regulation statement 26
9.2 Recruitment and consent 26
9.3 Benefits and risks assessment, group relatedness 26
9.4 Compensation for injury 27
10. ADMINISTRATIVE ASPECTS AND PUBLICATION 28
10.1 Data management and record keeping 28
10.2 Trial registration 28
10.3 Annual progress report 28
10.4 End of study report 28
11. REFERENCES 29

Summary of revision history

**Ref No**	**Date**	**Person**	**Version**	**Reason**
NLnavigation1.0	13-FEB-2013	K.F.D. Kuhlmann	1.0	
NLnavigation1.0	25-MAR-2013	K.F.D. Kuhlmann	2.0	revision
NLnavigation1.0	19-AUG-2013	K.F.D. Kuhlmann	3.0	revision
NLnavigation1.0	16-DEC-2013	K.F.D. Kuhlmann	4.0	revision
NLnavigation1.0	25-OCT-2014	J.A. Nijkamp	5.0	extension
NLnavigation1.0	22-SEP-2015	J.A. Nijkamp	6.0	extension
NLnavigation1.0	18-JAN-2016	J.A. Nijkamp	7.0	Extension
NLnavigation1.0	xxx	H.C. Groen	8.0	Extension

LIST OF ABBREVIATIONS AND RELEVANT DEFINITIONS

ABR	ABR form, General Assessment and Registration form, is the application form that is required for submission to the accredited Ethics Committee (In Dutch, ABR = Algemene Beoordeling en Registratie)
AE	Adverse Event
AR	Adverse Reaction
CA	Competent Authority
CCMO	Central Committee on Research Involving Human Subjects; in Dutch: Centrale Commissie Mensgebonden Onderzoek
CRLM	Colorectal liver metastases
CV	Curriculum Vitae
DSMB	Data Safety Monitoring Board
EU	European Union
EudraCT	European drug regulatory affairs Clinical Trials
GCP	Good Clinical Practice
IB	Investigator's Brochure
IC	Informed Consent
METC	Medical Research Ethics Committee (MREC); in Dutch: medisch ethische toetsing commissie (METC)
(S)AE	(Serious) Adverse Event
SPC	Summary of Product Characteristics (in Dutch: officiële productinfomatie IB1-tekst)
SUSAR	Suspected Unexpected Serious Adverse Reaction
Wbp	Personal Data Protection Act (in Dutch: Wet bescherming persoonsgevens)
WMO	Medical Research Involving Human Subjects Act (in Dutch: Wet Medisch-wetenschappelijk Onderzoek met Mensen

SUMMARY

Rationale:

Image-guided navigation surgery allows for full utilization of pre-operative imaging during surgery, and has the potential of reducing both irradical resections and morbidity. This is the first feasibility and safety registration study towards clinical implementation.

Objectives:

Primary: Feasibility and safety of an in-house developed electromagnetic navigation system during abdominal surgery.

Secondary: Evaluation of the accuracy of the system. During the procedure, possible improvements of the navigation hardware and software will be recorded and the handling during surgery will be evaluated. Extra time needed to use the navigation system during surgery will be recorded.

Study design:

An observational registration study.

Study population:

Patients undergoing scheduled for abdominal surgery by laparotomy. A total of 95 patients will be included in this study.

Intervention:

Extra contrast enhanced CT-scan of the pelvis and abdomen one day preoperatively. One extra intra-operative XperCT scan during surgery (no contrast). Identification of iliac vessels and ureters during surgery using an image-guided navigation probe. If a rigid tumour, or pathologic lymph nodes along the large vessels are present, the navigation system will be used to localize them.

Main study parameters/endpoints:

Feasibility, safety and handling of an electromagnetic navigation system during abdominal surgery.

Nature and extent of the burden and risks associated with participation, benefit and group relatedness:

Participation in the study will not involve additional visits to the hospital for the included patients. Patients will be informed for the study during the pre-operative outpatient clinic appointment. Informed consent will be obtained before or at admission to the hospital at least one day before operation.

One day before surgery, when patients are already admitted to the hospital, a contrast-enhanced CT-scan of the abdomen is performed. During the scan three removable electromagnetic reference-marker patches are placed on the skin at superficial bony

structures of the pelvis, such as the anterior superior iliac spine. The CT scan will be used to create a three-dimensional anatomical reconstruction of the organs at risk within the pelvis. Before surgery, the reference-markers will be positioned at the same locations. An intra-operative XPerCT scan will be used to assess the exact marker positions in the OR setup. The reference-markers will be used during surgery to correlate the CT scan to the actual patient position. During surgery, the iliac vessels and ureters will be identified using the guidance of a blunt tip probe of the navigation system. Total time of the proposed measurements, including intra-operative scan, will take no longer than 20 minutes. It is highly unlikely that the iliac vessels and ureters will be damaged during this procedure, since the navigation procedure is aimed at navigating towards the structures, without disrupting the patient's anatomy. When the tumour is rigid, e.g. sacral invasion, or when pathological lymph nodes are present outside the standard total mesorectal excision region, the navigation system will be used to localize these structures. The total burden for the patient will be one additional contrast enhanced CT scan, one XPerCT scan, and an extension of the total surgery procedure with no more than 20 minutes.

This is a registration study to evaluate the overall performance, safety and potential benefit of the surgical navigation system during abdominal surgery. The surgeons are aware of the experimental setup, and are therefore responsible for the navigation interpretation and actions. The eventual goal of this project is to judge the potential use of the navigation system for further clinical studies. When the system proves safe and accurate, further studies will be directed to improve tumor free surgical resection margins in abdominal surgery as well as to reduce the morbidity rate by more selective resections sparing more healthy tissue by intra-operative navigation.

1. INTRODUCTION AND RATIONALE

Clinical problem:

Standard treatment of rectal cancer consists of pre-operative (chemo-) radiation followed by surgical resection of the rectum. Resection can be challenging, especially in patients with large tumours, recurrent disease or tumours that have been treated with a long scheme of chemo-radiation. In these patients, anatomical planes are disturbed due to the tumour ingrowth, fibrosis or radiation effects. This can impede tumour free resection margins as well as the identification of surrounding structures (e.g. ureters and hypogastric plexus) and hence could result in damage of these structures. In distal rectal cancer, the number of resections that still contain tumour tissue at the resection edge mounts up to 30%. Better intra-operative guidance could improve these results significantly.

Damage to the ureters during surgery for pelvic tumours leads to reinterventions, long time morbidity and even perioperative mortality. The incidence of ureteric damage varies between 0.5 and 10% in most series and is highly dependent of the type and extend of the surgical intervention[1;2]. The incidence of damage to the hypogastric plexus approaches 30%. Damage of the hypogastric plexus results in bladder dysfunction (e.g. urine retention, stress or urge incontinence, loss of bladder sensitivity) and sexual disorders (e.g. erection and ejaculation disorders, decreased vaginal lubrication). Bladder dysfunction occurs in 20-30%, and sexual disorders in approximately 30% of patients after rectal surgery[3;4]. These complications have a significant impact on the patients' quality of life.

Integration of imaging during surgery

Pelvic tumours are diagnosed and judged on resectability using MRI and CT images. All the pre-operative information gathered from these scans is currently used during surgery by means of the "surgeon's brain", i.e. the surgeon reviews the information preoperatively and translates it into actions during surgery. The pre-operative images become more valuable when they are available during surgery and when the real-time positions of surgical tools can be related to the preoperative images during surgery. Fitted in a 3D environment, this would provide better assessment of resection planes and avoidance of vital structures. Image guided navigation systems can be used to integrate preoperative imaging in the surgical procedure.

Background of intra-operative navigation:

The continuously improving quality of medical imaging as well as the development of the supporting computer systems have led to the rise of image guided navigation in surgery. Pre- and per-operative acquired images (*e.g.* CT, MRI, ultrasound) are the key imaging modalities in navigation and are used to create three-dimensional road maps used for localization, orientation and guidance during surgery. In order to utilize the road map, a tracking system needs to be used which enables the correlation of the preoperative imaging to the actual position of the patient on the operation table. When implemented, the application of navigation during surgery results in three-dimensional insight and offers a view beyond the surfaces of organs. Furthermore, it can be used to reproduce pre-operative resection planning. This novel technique will be part of the operating room of the future and can lead to safer, tissue sparing and oncological more accurate procedures with an improved clinical outcome.

The application of image guided navigation is under investigation in many surgical fields. It is well established in surgical procedures of rigid structures such as bone and brain. Historically, neurosurgeons are the key players in this field and have already advanced to clinical studies to assess the effect of their navigation techniques[5]. Navigation in neurosurgery originated from frame-based stereotactic procedures and is nowadays generally applied in the treatment of gliomas and brain metastases [5;6]. In orthopedic surgery navigation is used in pelvic screw fixation, leading to reduced malposition rates, and in musculoskeletal tumor resection[7-10]. ENT (ear, nose and throat) and maxillofacial surgeons apply navigation techniques in various procedures such as posttraumatic craniofacial reconstruction of the skull and cochlear implantation[11;12].

The above mentioned fields of surgery are all based on the rather fixed positions of the bone and soft tissue components. Retroperitoneal organs, e.g. kidney, adrenal glands and iliac vessels can also be considered as relatively fixed and are therefore accessible targets for navigation surgery using preoperative images. Adrenalectomies were performed in a pilot setting using a laparoscopic navigation system[10]. Video overlay of reconstructed three-dimensional CT-images onto real-time laparoscopic footage was successfully tested in laparoscopic nephrectomies[13].

A technically more challenging field for surgical navigation is the abdomen. Organ movement, breathing motion and tissue deformation make the maintenance of accurate anatomical representation during the procedure very complex. More importantly, intra-abdominal targets such as colon or rectum tumors often do not have a fixed position. To accomplish real-time surgical navigation in the presence of “moving targets” a novel surgical tracking and navigation system needs to be developed.

There are two main technical options for surgical tracking systems. The first is optical tracking, which is based on a set of 2D or 3D camera's which can be used to detect active or passive markers on the patient and on surgical instruments. Secondly, electromagnetic (EM) tracking can be used, which is based on a magnetic field generator and EM-sensitive sensors. The position of a sensor in the magnetic field is associated with generation of a specific current, revealing the sensor position. Optical tracking systems are known for their accuracy, which is sub-millimeter, but they are dependent on a direct line-of-sight between the camera's and the markers in order to provide positional information. Due to the nature of magnetic fields, EM-navigation systems do not depend on a line-of-sight, while the systems have an accuracy of 1-2 mm. A further challenge for EM systems is the influence of ferromagnetic material on the stability of the generated magnetic field.

In our project we aim to develop a navigation system for colorectal cancer surgery which gives us real-time information on the position and orientation of the tumor. Simultaneously, the system will also be used to visualize and localize organs at risk with respect to the tumor. Since the tumor is not visible during the majority of surgical procedures, the use of optical tracking systems is limited. We are developing and implementing a surgical image-guided EM navigation system in which traceable sensors are implanted in or near the tumor to provide the surgeons with real-time information on the tumor location and orientation. Reference sensors, which are placed on the patient surface during acquisition of preoperative scans, are used during surgery to relate the imaging information to the actual patient position during surgery. Organs at risk which are relatively rigid with respect to the reference sensors, such as the iliac vessels and a large part of the ureters, can be located using the reference sensors. This is also valid for pathologic lymph nodes which are located near the large vessels, as well as for tumors which are rigid due to invasion of pelvic structures. The final system with implanted sensors in the tumor will provide accurate information on tumor position and extent for radical resections independent of the mobility of the tumor, and simultaneously, better insight in organs at risk for reduced morbidity.
The aim of this project is to gain first experience with the electromagnetic navigation system and its application during abdominal surgery. This first step encompasses the evaluation of the feasibility and safety of the system. Furthermore, the accuracy of the system to identify anatomical landmarks and structures with a relatively fixed position, such as iliac vessels, ureters, and fixed tumor deposits is assessed.

2. OBJECTIVES

The aim of this study is to gain experience using the electromagnetic navigation system for accurate identification of anatomical structures during abdominal surgery.

Primary Objective:

Feasibility and safety of an in-house developed electromagnetic navigation system during abdominal surgery.

Secondary Objectives:

Evaluation of the accuracy of the system. Evaluation of possible improvements of the navigation hardware and software, especially the handling in preparation towards and during surgery. Extra time needed to use the navigation system during abdominal surgery will be recorded.

Further clinical development and perspectives:

It is our aim to develop a fully integrated electromagnetic navigation system for colorectal cancer surgery. This system should provide accurate real-time information on the exact location and orientation of the tumour, the vital surrounding structures as well as the surgical instruments during the procedure. To provide real-time feedback of the location of the tumour, we are investigating the possibility of placing sensors in or around the tumour. However, as explained earlier, in the present project sensors patches will only be placed on the body surface of the patient. A secondary goal is to integrate this system in a laparoscopic environment. Image overlay techniques will be used to create a real-time virtual laparoscopic view where tumour, surrounding structures and instruments are superimposed over the regular laparoscopic view in the correct direction and dimensions. This results in a virtual environment where the surgeon is able to look beyond the surfaces of anatomical layers and organs.

3. STUDY DESIGN

General

The study is designed as an observational registration study.

Patients eligible for inclusion into this study are patients admitted to The Netherlands Cancer Institute – Antoni van Leeuwenhoek (NKI-AvL) and are planned to undergo scheduled abdominal surgery by laparotomy.

Procedures

- One day before surgery a contrast-enhanced CT-scan of the pelvis and abdomen is performed. During the scan three removable electromagnetic marker-patches are placed on superficial bony structures of the pelvis, such as the anterior superior iliac spine. Locations will be marked precisely with a permanent marker.
- Segmentations and three-dimensional reconstructions are made of the pelvic bone, the iliac vessels and ureters using the CT-scan
- The resulting 3D reconstructions are evaluated by the surgeon who will perform the surgical procedure.
- Before surgery, the marker-patches will be positioned at the scan locations and the navigation system will be calibrated. Patch positioning is checked in the safety procedure described below (5.2).
- The 3D reconstructions will be loaded into the navigation software.
- An intra-operative XPerCT (no contrast fluid used) is acquired to assess the actual patch positions with respect to the 3D model. The XPerCT is used to improve the navigation accuracy.
- A fast and robust safety procedure will be performed before application of the sterile field, to get an estimate of the registration and navigation accuracy. If tolerance limits are exceeded, the navigation procedure during surgery will be cancelled. The exact safety protocol is described in section 5.2.
- During the surgical procedure, the iliac vessels and ureters will be identified by the surgeon using the guidance of a blunt tip probe of the navigation system.
- Total time of the proposed measurements, including intra-operative scan, will take no longer than 20 minutes. Extra time will be recorded in this study.
- If pathologic lymph nodes or a rigid tumour is present in the patient, the location will be reviewed by means of the navigation system. This localization is not expected to increase the total surgery time, and will often even reduce surgery time.

4. STUDY POPULATION

4.1 Population (base)

Eligible patients are patients of the Netherlands Cancer Institute (NKI-AvL), who are scheduled for abdominal surgery by laparotomy. In the enrolment process the "patient informatie" will be used and the informed consent must be signed prior to the intervention.

4.2 Inclusion criteria

- Patients who are scheduled for abdominal surgery
- Locally advanced T3 or T4 tumour or local recurrent tumour
- Patients should be suitable for contrast enhanced CT scanning
- A signed informed consent
- Patients ≥ 18 years old

4.3 Exclusion criteria

- Patients with metal implants in the pelvic area
- Patients with a pacemaker

4.4 Sample size calculation

This project is a proof of principle study. The goal is to assess feasibility and safety of the electromagnetic navigation system in a clinical environment during surgery. The system is already extensively tested in a non-clinical setting and has shown to be highly accurate. Pre-clinical tests within the OR have shown that some environmental changes might influence the accuracy of the system. However, assessing all combinations of possible environmental changes is impossible. We have to perform this registration study to have an estimate of the expected environmental influences during surgery.

During the course of the study, we have identified an import application for navigation; i.e. locally advanced T3 or T4 rectal tumour and tumour recurrences in the abdomen and pelvic area. Lately, due to logistic reasons (OR planning), the drop-out of patients who signed informed consent was higher than expected. In order to obtain sufficient accuracy measurements to obtain statistical relevant numbers in this study, 20 more patients should be included. This accounts for 15 successful measurements and 30% chance of failure.

Inclusion of maximally 95 patients will be sufficient to evaluate and improve the system during abdominal surgery. No sample size calculation is performed.

5. TREATMENT OF SUBJECTS

5.1 Study procedure

All included patients are already scheduled for surgery. The programmed surgical procedure will not in any way be influenced by the measurements, other than an extension of total operating time by a maximum of 20 minutes, which is clinically acceptable in surgical procedures which generally take over 3 hours.

Specific study procedure:

Preparation:

- One day before surgery a contrast-enhanced CT-scan of the pelvis and abdomen is performed. During the scan three removable electromagnetic marker-patches are placed on the skin at superficial bony structures of the pelvis, such as the anterior superior iliac spine. Locations will be marked precisely with a permanent marker.
- Segmentations and three-dimensional reconstructions are made of the pelvic bone, the iliac vessels, ureters, the tumour, and pathologic lymph nodes using the CT-scan.
- The resulting 3D reconstructions are evaluated by the surgeon who will perform the surgical procedure.

In the OR:

- In the OR the patient will be placed on the table according to standard protocol, with the exception that the EM field generator will be placed between the table and the patient (Fig. 1).
- The marker-patches will be positioned at the scan locations and the navigation system will be calibrated.
- The 3D reconstructions will be loaded into the navigation software.
- An intra-operative XPerCT (no contrast fluid used) is acquired to assess the actual patch positions with respect to the 3D model. The XPerCT is used to improve the navigation accuracy.
- Before surgeons start applying the sterile field, an estimate of the registration accuracy between the CT and actual marker positions will be performed. If tolerance limits are exceeded, the navigation procedure during surgery will be cancelled.
- At the moment the laparotomy is advanced to the stage in which the iliac vessels are visible for the surgeon the study measurements are started.
- After the measurements the surgeon will proceed with the standard surgery. If the tumour is rigid, or pathologic lymph nodes are present around the large vessels, the navigation system can be used to localize them.

Actual measurements:

- First the surgeon will point the blunt tip probe at the bifurcation of the aorta into the left and right common iliac artery, and also both bifurcations into the internal and external iliac artery. These measurements will be performed without direct

visualization of the navigation system to the surgeon. For each pointed position the estimated position on the navigation system will be stored.

- In the second part, the surgeon will use the navigation system and the probe to localize the ureters. These are generally not visible for the surgeon. The resulting location of ureter tracts will be visually assessed by the surgeon in terms of trueness.

Total time of the proposed preparation and measurements during the surgical procedure will take no longer than 20 minutes. Extra time needed to use the navigation system during surgery will be recorded.

Specific participation for the patient in the study ends during the operation after the measurements have been performed.

5.2 Investigational products

The navigation system used for this study is composed of elements of the Aurora Electromagnetic Measurement System from Northern Digital Inc. (Waterloo, Ontario, Canada) and is combined with in-house developed software for the navigation and visualisation. The system is developed for use in the NKI-AvL only, and there is no current intent to commercialize the system. According to Dutch national guidelines (beleidsplan medische apparatuur), and agreements between the METC and the department of clinical physics, a tailored safety management and risk assessment protocol should be followed which is adapted to the specific medical device.

For technical validation of the navigation system before start of the study we will provide:

- A risk assessment, in collaboration with Michiel Sinaasappel (Clinical physicist)
- A detailed description of the medical device and software, together with a report of pre-clinical tests on system accuracy, in collaboration with Michiel Sinaasappel (Clinical physicist)
- A report on electrical safety, in collaboration with Michiel van der Meer (Electrical safety officer)
- A safety protocol for sterile use of the probe and reference markers in collaboration with Syb Beeksma (Sterile safety officer)
- A detailed operating manual for use in the OR, in collaboration with Marcel Meijer (Teamleader OR)

After all protocols and manuals have been approved, approval of Saar Muller (head of clinical physics department) will allow the use of the navigation system for the study.

The major risk domains for the current study are electrical safety, sterility, and unforeseen inaccuracies leading to misguidance of the surgeon.

The NDI Aurora hardware has been embedded in several commercial navigation systems, such as the Philips Percunav system. Electrical safety is extensively tested and described by NDI, and will be independently validated by Michiel van der Meer.

Two components of the system will come in contact with the patient, being the reference patches and the blunt tip probe. The reference patches are developed by Philips in collaboration with NDI, and are provided in a sterile package. The blunt tip probe can withstand autoclave procedures, and will be tested on sterility by Syb Beeksma. An alternative option is to use a sterile cover which is also used for hand-held ultrasound probes.

Inaccuracies of the system can guide the surgeon into the wrong anatomic direction. This could lead to unwanted disruption of anatomy. In this study, the navigation system will not be used to blindly guide the surgeon, and only visible, approachable regions will be localized and pointed without disruption of anatomy. This minimizes the risks for this study. Furthermore, a quick and reliable safety and accuracy test will be performed before start of surgery. If there are any signs of major inaccuracies, the navigation procedure during surgery will be cancelled. The safety procedure is described at the end of this paragraph.

Navigation hardware

The navigation system consists of several components, being 1) the EM field generator; 2) the blunt tip trackable probe; 3) the reference marker-patches; 4) the sensor interface units; 5) the system control unit.

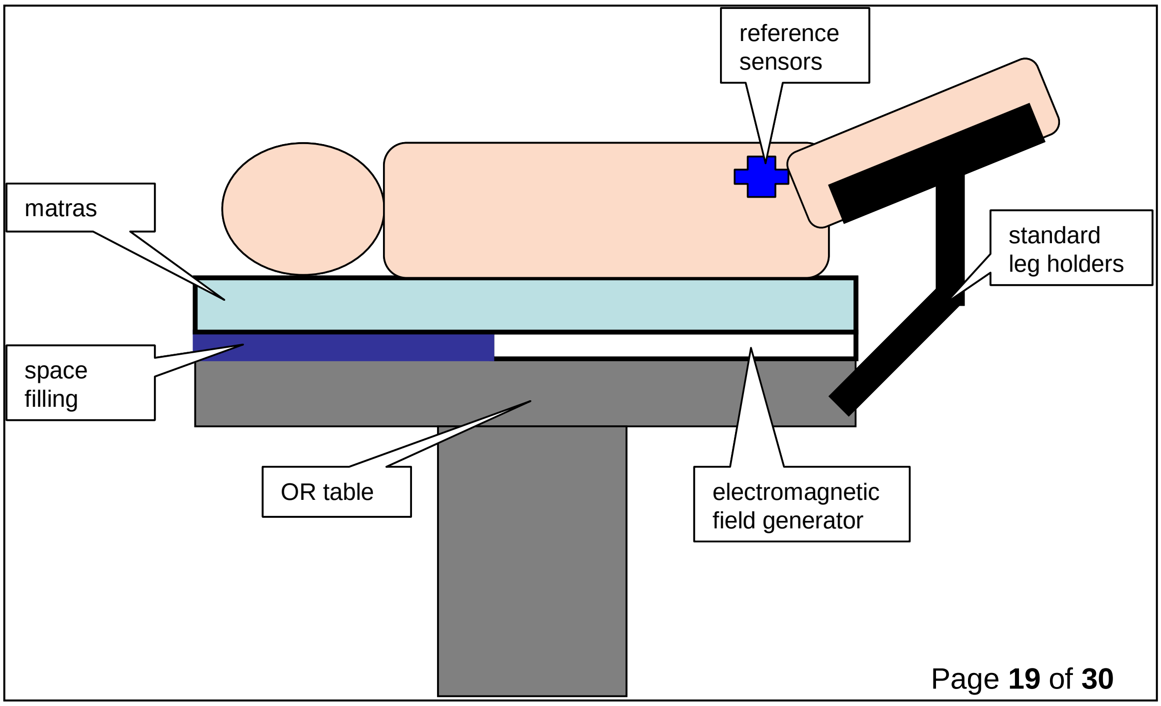

Figure 1: Schematic side-view of a typical patient setup for pelvic cancer surgery, combined with the Aurora navigation system

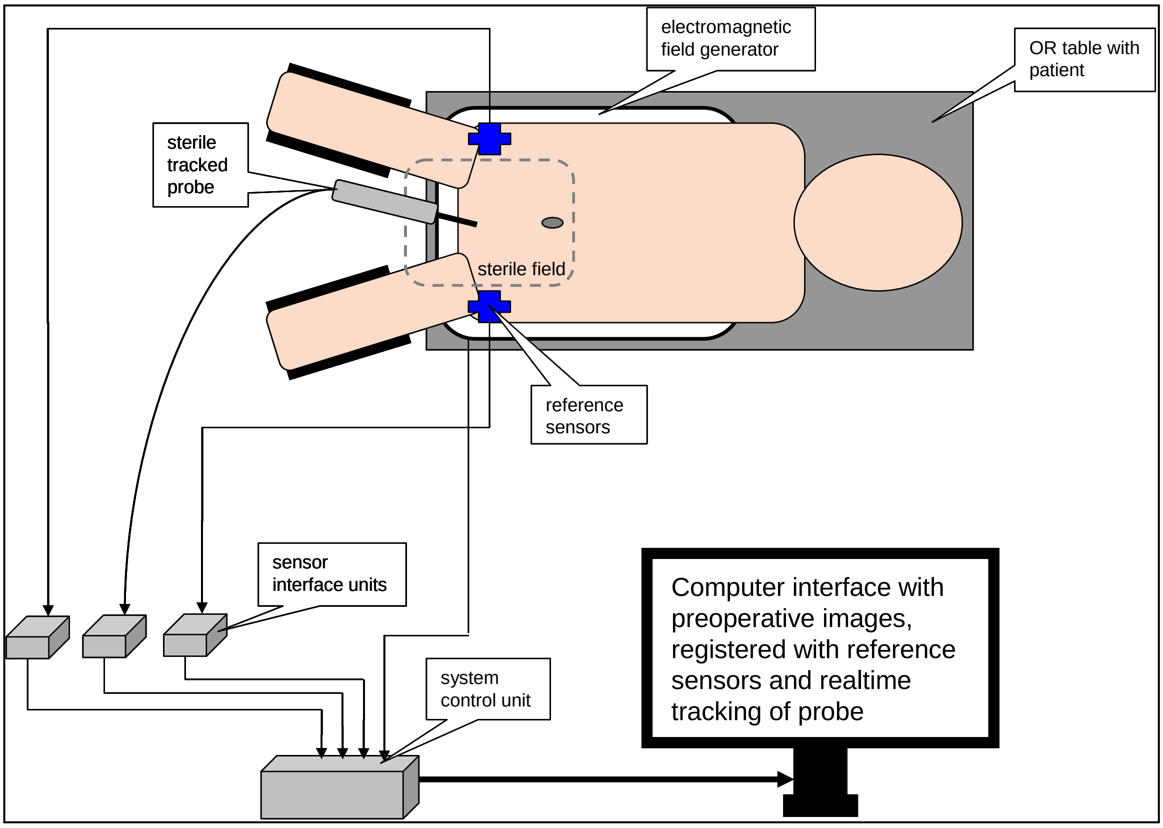

Figure 2: Overview of the navigation hardware components and the interconnections between the components

A side-view of the patient setup in combination with the navigation system is shown in figure 1. A schematic overview of the navigation hardware and the interconnections is shown in figure 2. Of the system components, the blunt tip probe and reference patches will come in contact with the patient. Only the blunt tip probe (Fig. 3) will actually be used within the sterile surgical field.

The EM navigation system generates a known electromagnetic field. Small EM sensors which are located within the EM field will generate a current which can be measured through a sensor interface. Based on the current the exact location and orientation of the sensor within the EM field can be determined. The EM sensors are typically 1 mm in diameter and 8-10 mm long. Sensors can easily be embedded in surgical tools, such as a blunt tip probe, a laparoscopic camera, or a surgical knife.

The used EM field generator is a next generation tabletop version. This generator is placed underneath the patient, and has a shielding layer to minimize influence of metal in the table

to the accuracy of the magnetic field. Maier-Hein *et al.* have shown that this EM field generator has an accuracy in the order of 1mm within a clinical environment, which is suitable for our application[14].

Blunt tip probe

This is a general purpose digitizing probe with a rigid, straight metal tip (Fig. 3).

Dimensions of the tip: 65 mm long x 3 mm diameter.

The probe will be sterilized via autoclave before every intervention.

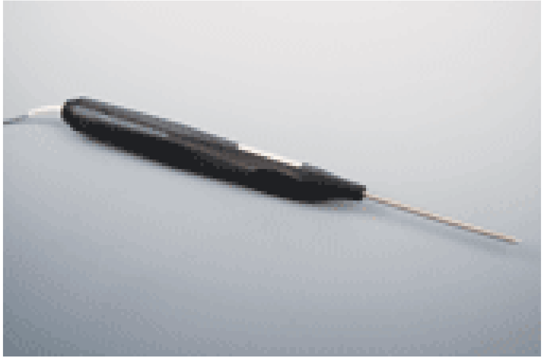

Figure 3: Aurora standard straight tip 6DOF Probe

Computer with navigation software

The navigation software is in-house developed. For the study a code freeze will be performed to formalize a software release. In collaboration with the clinical physics department (Michiel Sinaasappel) the software will be tested on consistency and reliability, and documentation will be stored. The system will be tested in a black-box approach, where an entire chain-test from acquiring CT data with reference markers until the actual navigation to known positions in a phantom will be tested. The software will be installed on a OR-compatible computer with HEPA filters for in-room use.

Safety protocol

To prevent delays and minimize the impact of the study procedures in clinical practice the following tests will be performed before the start of surgery. When the reference patches have been applied on the patients' skin in the OR, the navigation system is started. Each patch consists of two sensors, resulting in a total of six sensors. The first step is to make a registration between the reference patch positions in the CT scan and the actual positions in the OR according to the navigation system. This is an automatic registration which takes less than one second. The resulting registration is quantified by assessment of the distances between the scanned and actual sensor positions after registration, where distances of 0 cm

are ideal. This is expressed in a root-mean-square (RMS) parameter, which is a general measure for registration accuracy. The distances are also provided for each sensor separately, to be able to localize and solve individual sensor misplacement. Each sensor should have a registration error smaller than 2.0 cm. If this level is exceeded, sensors can be taken out of the registration. A minimum of four remaining sensors should be used in the final registration. The final RMS of the registration should be within 1.0 cm. If this level is exceeded the procedure will be cancelled.

To further validate the system performance, the blunt tip probe will be used to point at each reference sensor. On the navigation screen the tip of the probe should visually be within 0.5 cm of each scanned reference sensor. Finally, as independent check, the probe is used to point at the belly button, which is then visually checked on the navigation system. If discrepancies of over 1.0 cm exist a quick assessment of possible accuracy improvements is done. If discrepancies remain, the navigation procedure during surgery is cancelled.

5.3 Use of co-intervention

No specific restrictions for included patients are specified. Included patients will follow the same pre-operative workup as non-included patients scheduled for a similar operation.

6. METHODS

6.1 Main study parameters/endpoints

During the surgical procedure, iliac vessels and ureters will be identified using the probe. To have an objective measure of accuracy, three locations on the iliac vessel tract will be pointed out by the surgeon without visualization of the navigation system. These points will be the bifurcation of the common iliac artery into the left and right artery, and also both bifurcations into the internal and external iliac artery. The estimated distance of the probe with respect to the bifurcations in the navigation system will be used as accuracy measure. During surgery, the surgeon will search for the ureters and expose them if needed for the surgical procedure. Navigation software and the probe will be used to estimate the tracts of the ureters within the patient. For several tracked positions the actual probe position will be stored in combination with an actual picture of the real situation. The visual measurements in this phase will also be used for assessment of the software usability. Rigid tumours and pathologic lymph nodes along the large vessels will be localized to gain insight in the use of the current navigation system with only external tracking markers. This experience will be used to design a proper clinical study. The addition of an intra-operative XPerCT scan is

expected to improve the navigation accuracy. To assess the improvement, we will register the external patches using both the position from the pre-operative CT scan, as well as the position from the XPerCT scan.

<u>Main endpoint</u>

In this study we aim to evaluate the feasibility and safety of the navigation system by identifying the iliac vessels and ureters.

6.2 Randomisation, blinding and treatment allocation

This is an observational study. All included patients will be subjected to the same procedure, therefore no randomization is required.

6.3 Withdrawal of individual subjects

Subjects can leave the study at any time for any reason if they wish to do so without any consequences. The investigator can decide to withdraw a subject from the study for urgent medical reasons.

If any technical or physical problems occur during the measurement procedure, prohibiting the anticipated measurements, the patient will be withdrawn from the study. This will not affect the scheduled operation.

6.4 Premature termination of the study

Reasons for premature termination of the study are:

- Occurrence of any serious adverse events that can in anyway be related to the study.
- Technical problems that prevent measurements to be performed.

6.5 Monitoring and auditing

There will be no planned monitoring or auditing visits scheduled.

7. SAFETY REPORTING

7.1 Section 10 WMO event

In accordance to section 10, subsection 1, of the WMO, the investigator will inform the subjects and the reviewing accredited METC if anything occurs, on the basis of which it appears that the disadvantages of participation may be significantly greater than was foreseen in the research proposal. In this case the study will be suspended pending further review by the accredited METC.

7.2 Adverse and serious adverse events

Based on the minimal extent of the planned intervention, no SAE's are expected for this study.

Adverse events, AE, are defined as any untoward medical occurrence, unintended disease or injury, or untoward clinical signs (including abnormal laboratory findings) in subjects, users or other persons, whether or not related to the investigational medical device.

All adverse events reported spontaneously by the subject or observed by the investigator or his staff will be recorded.

A serious adverse event, SAE, is an adverse event that:

a) led to death,

b) led to serious deterioration in the health of the subject, that either resulted in

1) a life-threatening illness or injury, or

2) a permanent impairment of a body structure or a body function, or

3) in-patient or prolonged hospitalization, or

4) medical or surgical intervention to prevent life-threatening illness or injury or permanent impairment to a body structure or a body function,

c) led to fetal distress, fetal death or a congenital abnormality or birth defect

All SAEs will be reported through the web portal *ToetsingOnline* to the accredited METC that approved the protocol, within 15 days after the investigator has first knowledge of the serious adverse reactions.

SAEs that result in death or are life threatening should be reported expedited to the METC. The expedited reporting will occur not later than 7 days after the responsible investigator has first knowledge of the adverse reaction. This is for a preliminary report with another 8 days for completion of the report.

If serious adverse effects come up that are related or can in any way be related to the study, the study will terminate.

7.3 Follow up of adverse and serious adverse events

All adverse events will be followed up until they have abated, or until a stable situation has been reached. Depending on the event, follow up may require additional tests or medical procedures as indicated, and/or referral to the general physician or a medical specialist.

7.4 Data Safety monitoring board (DSMB)

Not applicable.

8. STATISTICAL ANALYSIS

8.1 Descriptive statistics

In this registration study, distances will be measured between the estimated probe positions with respect to the bifurcation of the common iliac artery into the left and right artery, both bifurcations into the internal and external iliac artery and ureters. Measurements will be compared within patients to assess patient specific systematic errors, and between patients to assess system specific systematic errors. Due to the nature of a registration study, significance can not be estimated. However, usability and subjective assessment of experience by the surgeons will be valuable for future projects. Assessment of the ureter tracts will be based on the surgeon's opinion, and scored in terms of excellent, reasonable, dubious or wrong. Actual assessment of tumour boundaries and suspected lymph node locations will be recorded and cross-referenced with pathology

ETHICAL CONSIDERATIONS

8.2 Regulation statement

The study will be conducted according to the principles of the Declaration of Helsinki (adopted during 59th WMA General Assembly, Seoul, October 2008) and in accordance with the Medical Research Involving Human Subjects Act (WMO) and other guidelines, regulations and Acts. Local METC approval and CE-certification of the blunt probe will be obtained prior to the start of the study.

8.3 Recruitment and consent

Patients eligible for the study will be contacted during the pre-operative outpatient clinical appointment by the investigator or treating physician. Written informed consent will be obtained at least 1 day prior to the scheduled operation, if patients approve to inclusion. See appendix 2, Patiënteninformatie en informed consent.

8.4 Benefits and risks assessment, group relatedness

Because of the nature of this study, we do not expect any adverse events to occur that are related to the intervention. Measurements shall only be performed during the scheduled operation under full anesthesia and physical monitoring.

Collected data will not be provided to surgical physicians. Moreover, planned surgical procedure will not be influenced by the measurements and hence patient treatment will not

be influenced by the measurements. Subjects who participate in the test will not benefit from the test nor experience unacceptable additional discomfort.

8.5 Compensation for injury

The investigator has a liability insurance which is in accordance with article 7, subsection 6 of the WMO.

The sponsor has both a “General and Product Liability Insurance” and a “Clinical Trial Insurance”, which is in accordance with the legal requirements in the Netherlands (Article 7 WMO and the Measure regarding Compulsory Insurance for Clinical Research in Humans of 23rd June 2003). These insurances provides cover for damage to research subjects through injury or death caused by the study.

1. € 450.000,-- (i.e. four hundred and fifty thousand Euro) for death or injury for each subject who participates in the Research;
2. € 3.500.000,-- (i.e. three million five hundred thousand Euro) for death or injury for all subjects who participate in the Research;
3. € 5.000.000,-- (i.e. five million Euro) for the total damage incurred by the organisation for all damage disclosed by scientific research for the Sponsor as ‘verrichter’ in the meaning of said Act in each year of insurance coverage.

The insurance applies to the damage that becomes apparent during the study or within 4 years after the end of the study.

9. ADMINISTRATIVE ASPECTS AND PUBLICATION

9.1 Data management and record keeping

Patient data will be handled confidentially and an identification code will be used to link the data to the subject. The principal investigator of the NKI safeguards the key to the code. In particular, for this study K.F.D. Kuhlmann, J. Nijkamp and T. Ruers will have access to the patient data. The handling of personal data complies with the Dutch Personal Data Protection Act (in Dutch: De Wet Persoonsbescherming). The signed informed consent statements, the listing of the identities of the patients, originals of the CRF's, and data collected during the study will be stored at the NKI.

9.2 Trial registration

Prior to initiation, the study will be submitted to the Netherlands National Trial register, which is a recognized and accepted by the World Health Organization and International Committee of Medical Journal Editors (ICMJE).

9.3 Annual progress report

The investigator will submit a summary of the progress of the trial to the accredited METC once a year. Information will be provided on the date of inclusion of the first subject, numbers of subjects included and numbers of subjects that have completed the trial, serious adverse events/ serious adverse reactions and other problems.

9.4 End of study report

The sponsor will notify the accredited METC of the end of the study within a period of 8 weeks. The end of the study is defined as the end of the last patient's procedure.

Within one year after the end of the study, the investigator will submit a final study report with the results of the study, including any publications/abstracts of the study, to the accredited METC.

10. REFERENCES

Reference List

(1) Kehinde EO, Al-Awadi KA, Al-Hunayan A, Okasha GH, Al-Tawheed A, Ali Y. Morbidity associated with surgical treatment of ureteric calculi in a teaching hospital in Kuwait. Ann R Coll Surg Engl 2003; 85(5):340-346.

(2) Hughes ES, McDermott FT, Polglase AL, Johnson WR. Ureteric damage in surgery for cancer of the large bowel. Dis Colon Rectum 1984; 27(5):293-295.

(3) Sterk P, Shekarriz B, Gunter S, Nolde J, Keller R, Bruch HP et al. Voiding and sexual dysfunction after deep rectal resection and total mesorectal excision: prospective study on 52 patients. Int J Colorectal Dis 2005; 20(5):423-427.

(4) Del RC, Sanchez-Santos R, Oreja V, De OJ, Biondo S, Pares D et al. Long-term urinary dysfunction after rectal cancer surgery. Colorectal Dis 2004; 6(3):198-202.

(5) Kubben PL, Ter Meulen KJ, Schijns OE, Ter Laak-Poort MP, van Overbeeke JJ, Santbrink H. Intraoperative MRI-guided resection of glioblastoma multiforme: a systematic review. Lancet Oncol 2011; 12(11):1062-1070.

(6) Senft C, Ulrich CT, Seifert V, Gasser T. Intraoperative magnetic resonance imaging in the surgical treatment of cerebral metastases. J Surg Oncol 2010; 101(5):436-441.

(7) Zwingmann J, Konrad G, Kotter E, Sudkamp NP, Oberst M. Computer-navigated iliosacral screw insertion reduces malposition rate and radiation exposure. Clin Orthop Relat Res 2009; 467(7):1833-1838.

(8) So TY, Lam YL, Mak KL. Computer-assisted navigation in bone tumor surgery: seamless workflow model and evolution of technique. Clin Orthop Relat Res 2010; 468(11):2985-2991.

(9) Wu K, Webber NP, Ward RA, Jones KB, Randall RL. Intraoperative navigation for minimally invasive resection of periarticular and pelvic tumors. Orthopedics 2011; 34(5):372.

(10) Lango T, Tangen GA, Marvik R, Ystgaard B, Yavuz Y, Kaspersen JH et al. Navigation in laparoscopy--prototype research platform for improved image-guided surgery. Minim Invasive Ther Allied Technol 2008; 17(1):17-33.

(11) Aschendorff A, Maier W, Jaekel K, Wesarg T, Arndt S, Laszig R et al. Radiologically assisted navigation in cochlear implantation for X-linked deafness malformation. Cochlear Implants Int 2009; 10 Suppl 1:14-18.

(12) Bell RB, Markiewicz MR. Computer-assisted planning, stereolithographic modeling, and intraoperative navigation for complex orbital reconstruction: a descriptive study in a preliminary cohort. J Oral Maxillofac Surg 2009; 67(12):2559-2570.

(13) Su LM, Vagvolgyi BP, Agarwal R, Reiley CE, Taylor RH, Hager GD. Augmented reality during robot-assisted laparoscopic partial nephrectomy: toward real-time 3D-CT to stereoscopic video registration. Urology 2009; 73(4):896-900.

(14) Maier-Hein L, Franz AM, Birkfellner W, Hummel J, Gergel I, Wegner I et al. Standardized assessment of new electromagnetic field generators in an interventional radiology setting. Med Phys 2012; 39(6):3424-3434.
