# Supplementary material for: Association of Image-Guided Navigation With Complete Resection Rate in Patients With Locally Advanced Primary and Recurrent Rectal Cancer: A Nonrandomized Controlled Trial
Source: JAMA Netw Open. 2020 Jul 8;3(7):e208522. doi: 10.1001/jamanetworkopen.2020.8522 (PMC7344384; doi:10.1001/jamanetworkopen.2020.8522)
Supplement: Supplement 2. — eFigure. Mean Scores of Items in the System Usability Scale [file jamanetwopen-3-e208522-s002.pdf]

Supplementary Online Content

Kok END, van Veen R, Groen HC, et al. Association of image-guided navigation with complete resection rate in patients with locally advanced primary and recurrent rectal cancer: a nonrandomized controlled trial. *JAMA Netw Open.* 2020;3(6):e208522. doi:10.1001/jamanetworkopen.2020.8522

eFigure. Mean Scores of Items in the System Usability Scale

This supplementary material has been provided by the authors to give readers additional information about their work.

©2020 Kok END et al. *JAMA Network Open*

eFigure. Mean Scores of Items in the System Usability Scale

For high usability, questions 1, 3, 5, 7, and 9 (dark gray) should be given higher scores, and questions 2, 4, 6, 8, and 10 (light gray) should be given lower scores. For each odd-numbered question, subtract 1 from the score; for each even-numbered question, subtract 5. Multiply the sum of the scores by 2.5 to obtain the overall score. In this study's questionnaire, the total score was 75 points.
